# miRNAs in anti-cancer drug resistance of non-small cell lung cancer: Recent advances and future potential

**DOI:** 10.3389/fphar.2022.949566

**Published:** 2022-10-25

**Authors:** Hang Yan, Shengjie Tang, Shoujun Tang, Jun Zhang, Haiyang Guo, Chao Qin, Haiyang Hu, Chuan Zhong, Li Yang, Yunhe Zhu, Haining Zhou

**Affiliations:** ^1^ Department of Thoracic Surgery, Suining Central Hospital, An Affiliated Hospital of Chongqing Medical University, Suining, China; ^2^ Graduate School, Institute of Surgery, Zunyi Medical University, Zunyi, China; ^3^ Graduate School, Institute of Surgery, Chengdu University of TCM, Chengdu, China

**Keywords:** drug resistance, miRNA, NSCLC, therapy, biomarker

## Abstract

Non-small cell lung cancer (NSCLC) is one of the most common malignant tumors worldwide. Clinical success is suboptimal owing to late diagnosis, limited treatment options, high recurrence rates, and the development of drug resistance. MicroRNAs (miRNAs), a range of small endogenous non-coding RNAs that are 22 nucleotides in length, have emerged as one of the most important players in cancer initiation and progression in recent decades. Current evidence has revealed the pivotal roles of miRNAs in regulating cell proliferation, migration, invasion, and metastasis in NSCLC. Recently, several studies have demonstrated that miRNAs are strongly associated with resistance to anti-cancer drugs, ranging from traditional chemotherapeutic and immunotherapy drugs to anti-vascular drugs, and even during radiotherapy. In this review, we briefly introduce the mechanism of miRNA dysregulation and resistance to anti-tumor therapy in NSCLC, and summarize the role of miRNAs in the malignant process of NSCLC. We then discuss studies of resistance-related miRNAs in chemotherapy, radiotherapy, targeted therapy, immunotherapy, and anti-vascular therapy in NSCLC. Finally, we will explore the application prospects of miRNA, an emerging small molecule, for future anti-tumor therapy. This review is the first to summarize the latest research progress on miRNAs in anti-cancer drug resistance based on drug classification, and to discuss their potential clinical applications.

## Introduction

Lung cancer is the most frequent cause of cancer-related death worldwide, with 2.09 million new cases and 1.76 million deaths estimated in the GLOBOCAN 2018 databases (Siegel et al., 2019; Siegel et al., 2021). It has been pathologically subdivided into two subsets: small-cell lung cancer (SCLC) and non-small cell lung cancer (NSCLC). NSCLC accounts for approximately 85% of all lung cancer cases, of which the most common types are lung adenocarcinoma (LUAD) and lung squamous cell carcinoma (LUSC) (Herbst et al., 2018). Generally, NSCLC is not detected and diagnosed until advanced-stage disease or symptomatic is present (Siegel et al., 2021). Cough is the most common symptom in 50–75 percent of patients, and is sometimes accompanied by chest pain, hemoptysis and dyspnea (Kocher et al., 2015). Surgery, radiotherapy, and chemotherapy are the three main treatments for lung cancer (Duma et al., 2019). In recent years, molecular targeted therapies and immunotherapy have become increasingly popular for the treatment of lung cancer (Hirsch et al., 2017). Research has shown that anti-angiogenic therapy has become a critical treatment method for NSCLC (Hu et al., 2020). Despite the clinical availability of multiple treatments for NSCLC, the 5-year survival rate remains dismal, largely owing to the emergence of resistance to therapies (Siegel et al., 2021). Drug resistance presents a significant obstacle in the treatment of NSCLC and conduce to disease progression, tumor recurrence, and greatly increased cancer mortality (Sarkar et al., 2010). The development of new therapeutic regimens for NSCLC has been restricted by a lack of reliable diagnostic and prognostic biomarkers and limited research on mechanisms that promote rapid cancer progression, early metastatic spread, and drug resistance.

**FIGURE 1:**
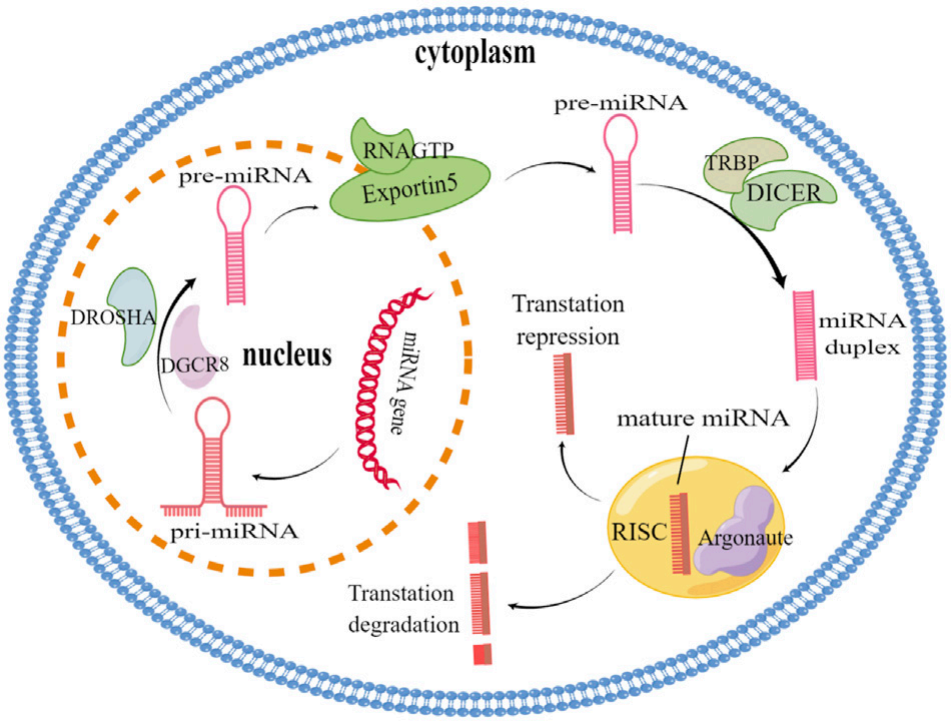
miRNA biogenesis and action. miRNA genes are transcribed by RNA polymerase II to produce primary miRNA (Pri-miRNA), Pri-miRNA is further processed by Drosha-Dgcr8 in the nucleus to form pre-miRNA, which is then exported to the cytoplasm *via* Exportin5/Ran GTP. TRBP, and DICER in the cytoplasm further process the pre-miRNA into mature short double strand RNA fragments. One mature miRNA is associated with the Argonaute protein (AGO2) and binds to the 3′UTR of the complementary site target mRNA of RISC leading to translation inhibition while the other strand is degraded.

MicroRNAs (miRNAs) are 18–25 nucleotides in length and function in gene expression and post-transcriptional regulation (Bartel, 2004). They are transcribed under the function of RNA polymerase II to generate miRNA primary miRNA (Pri-miRNA), which is processed by Drosha protein in the nucleus as precursor miRNA (pre-miRNA). The pre-miRNAs are subsequently transported to the cytoplasm in exportin-5 mediated, and further processed by Dicer enzyme into mature miRNAs in the cytoplasm. Then, a mature miRNA strand is incorporated into the RNA-induced silencing complex (RISC) (Figure 1). By this way, the protein complex can complement the target mRNA; Binding to the complementary sequence in the 3′ untranslated region (3′-UTR) of the target mRNA and the target gene was silenced by either degrading mRNA or blocking the translation of mRNA (Bartel, 2004; Kim, 2005; Winter et al., 2009; Krol et al., 2010; Jonas and Izaurralde, 2015). Through the post-transcriptional regulation of gene expression, miRNAs are participate in all kinds of cellular processes, including proliferation of cell or apoptosis, tumorigenesis or invasion–metastasis, angiogenesis and cancer progression (Lu et al., 2005; Shivdasani, 2006; Lin and Gregory, 2015). In recent years, miRNAs have been shown to have tremendous effects on tumor progression (Croce, 2009). Interestingly, miRNAs are refer to drug resistance in many cancers, and their dysregulation has been highlighted as an emerging mechanism of drug resistance (Zhang and Wang, 2017; Wei et al., 2019a). Understanding the relationship between miRNAs regulation and drug resistance mechanism is a new direction of anti-tumor drug resistance.

The effect and expression status of miRNAs have been extensively studied in lung cancer tissues and cell lines (Iqbal et al., 2019). MiRNAs in lung cancer tissues and tumor cells and their mechanisms of action in tumorigenesis (include invasion, metastasis, recurrence, drug resistance, etc.) are hotly reported. In this review, we briefly summarize the current understanding of the miRNAs link with the progression of NSCLC, discussing their potential in the treatment of NSCLC.

## Function of MicroRNAs in progression of NSCLC

Sustained cell proliferation is a fundamental characteristic of cancer, which is regulated by miRNAs bioactivities. For example, decreased expression of the let -7 miRNA family is common in lung cancer patients because let -7 directly or indirectly inhibits multiple target genes involved in cell cycle and cell division (Osada and Takahashi, 2011). Johnson and colleagues reported evidence that let-7 expression is associated with lung cancer proliferation, suggesting that let-7 deletion significantly enhances cell division in the S-phase of the A549 cell cycle (Johnson et al., 2007). Moreover, NSCLC tissues are markedly up-regulated by miR-196b-5p, which directly targets the tumor suppressors GATA6 and TSPAN12 to promote lung cancer cell proliferation and cell cycle (Liang et al., 2020). In contrast, Harel et al. reported that exosome miR-512 stopped the proliferation of lung tumor cells by targeting member four of the TEA domain family (TEAD4), indicating that miR-512 has the function of tumor inhibition (Adi Harel et al., 2015). Additionally, the upstream Ras signaling pathway molecule SOS2 was inhibited by miR-148A-3p *in vitro*, leading to inhibition of NSCLC cell proliferation (Xie et al., 2019). Besides, it has been reported that miR-520A-3p is involved in the downstream PI3K/Akt/mTOR pathway, thereby affecting cell proliferation (Lv et al., 2018). To further understand the important relationship between miRNA and lung cancer cell proliferation is still the focus of current experimental studies.

Evasion of programmed cell death (apoptosis) is another essential hallmark of cancer, with external and internal pathways predominating. Similarly, studies have shown that miRNAs can take part in the regulation of lung cancer cell apoptosis through acting on transcription factors involved in endogenous and exogenous pathways. The intrinsic pathway is apoptosis caused by dysfunction of some members of the BCL-2 family. Wang et al. reported that miR-16–1 induces apoptosis by downregulating Bcl-2 (Wang et al., 2016). Tian et al. showed that miR-130b indirectly upregulates Bcl-2 *via* the peroxisome proliferator-activated receptor gamma (PPAR-γ)/VEGF pathway and suppresses lung cancer cell apoptosis (Tian et al., 2016). The extrinsic pathway refers to apoptosis that initiated start with extracellular death-inducing signals *via* cell surface receptors (e.g., TNF family receptors). Garofalo et al. reported that a trail-targeted molecular signaling cascade initiated by Mir-221/222 resulted in loss of PTEN signaling, which in turn activated the PI3K/AKT/mTOR pathway, showing a strong inhibitory effect on TRAIL-mediated apoptosis (Garofalo et al., 2009). Subsequently, the researchers also found that miR-760 was detected to play a key role in TRAIL-induced apoptosis of lung cancer cells (Zhang X.et al., 2018). Overall, much remains to be learned about the potential role of miRNAs in apoptosis activity of lung cancer cells.

The invasion–metastasis cascade is a distinct cancer behavior, and miRNAs are not absent from the process. Recent studies have verify that miR-31-3p have the function of facilitates proliferation, invasion, and migration of NSCLC cells by targeting FOXO1 (Zeng et al., 2022). In addition, the study of Yang et al. showed that miR-1246 modulates the Wnt/β-catenin pathway by targeting GSK-3β/β-catenin which promotes the invasion and metastasis of lung cancer tumors (Yang et al., 2019). Epithelial-to-mesenchymal transition (EMT) is the central process to cancer metastasis, whose feature are the loss of E-cadherin-mediated cell adhesion, increase in cell viability and promoting tumor aggressiveness and metastasis (Du and Pertsemlidis, 2010). However, miR-200 targets zinc finger E-box-binding homeobox (ZEB)1 and ZEB2, and also encode transcriptional repressors of E-cadherin. Therefore, the increased expression of E-cadherin is caused by the up-regulation of miR-200, resulting in decreased vitality of lung cancer cells and inhibition of tumor metastasis (Chen L.et al., 2014). Additionally, the miR-183–96–182 cluster also inhibits invasion and metastasis of lung cancer by targeting Foxf2 (Kundu et al., 2016). Therefore, the study of the association between miRNAs and lung cancer metastasis may be a meaningful focus for clinical prediction of lung cancer recurrence.

Neovascularization is an essential condition for cancer survival and the effect of vascular endothelial growth factor (VEGF) on angiogenesis induction is irreplaceable (Hanahan and Weinberg, 2011; Nowak-Sliwinska et al., 2018). Many miRNAs have been reported to be involved in regulating angiogenesis in various cancer cell lines (Wang et al., 2018). Coincidentally, inhibition of angiogenesis by targeting VEGF by members of the miR-200 family has been reported (Pecot et al., 2013). Hu et al. (2014) agreed that upregulation of miR-126 and miR-128 could directly targeting VEGF-A and VEGF-C showed the capacity of inhibiting angiogenesis in lung tumor cell lines. In contrast, exosome miR-25-3p regulates the VEGFR2 and ZO-1 expression of endothelial cells by targeting KLF2 and KLF4, thereby promoting vascular permeability and accelerating angiogenesis (Zeng et al., 2018). In hypoxia, miR-23a expression in lung-derived exosomes induces HIF-1 α by targeting prolyl hydroxylase 1 and 2 (PHD1 and 2), thereby activating the VEGF pathway and promoting angiogenesis (Hsu et al., 2017). Moreover, through RT-qPCR analysis, Chen et al. revealed that silencing miR-511-5p increases the mRNA expression levels of VEGF-A, which promotes the proliferation of vascular endothelial cells (Chen et al., 2020a).

The examples mentioned above are sufficient to prove that miRNAs can be used as new regulatory factors in tumor genesis and development. In-depth study on the relationship between miRNAs and proliferation, apoptosis and metastasis of lung cancer cells is expected to become a new biomarker for diagnosis, treatment and prognosis of lung cancer.

## MicroRNAs and drug resistance in NSCLC

**FIGURE 2:**
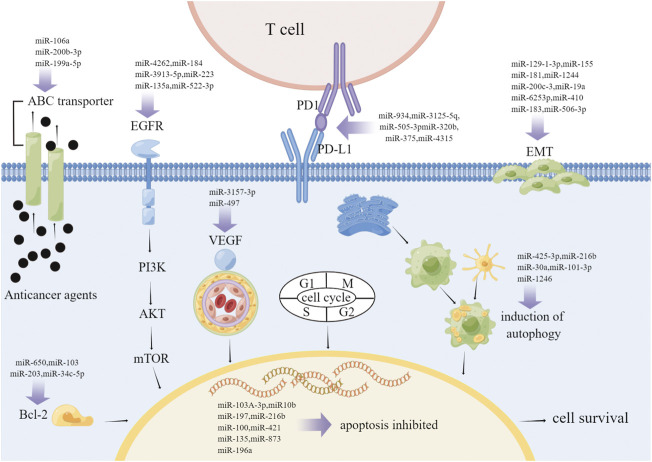
Drug resistance in non-small cell lung cancer. The mechanisms of drug resistance in NSCLC are complex and diverse, including ABC transporter activity, anti-apoptosis, autophagy induction, EGFR mutation, EMT promotion, angiogenesis promotion and checkpoint changes. The figure shows the mechanism of miRNA involved in different drug resistance processes of NSCLC. Abbreviations: ABC, ATP-binding cassette; EMT, epithelial–mesenchymal transition; EGFR, epidermal growth factor receptor; VEGF, vascular endothelial growth factor.

Although a variety of therapeutic approaches like chemotherapy, targeted therapy and radiotherapy have been used clinically, drug resistance remains a major impediment to effective NSCLC therapy. However, the expression of miRNAs has also been implicated with the booming of drug resistance in NSCLC, such as controlling cell proliferation, inhibiting apoptosis and activating autophagy (Lampis et al., 2020). Many studies have reported an association between miRNAs and drug resistance in NSCLC. For example, platinum-based therapy is the preferred regimen for NSCLC, and cisplatin sensitivity is increased at an unstoppable speed in NSCLC cell lines with miR-106b upregulation (Yu et al., 2017). Both miR-146b and miR-218 decreased the resistance of NSCLC cells to cisplatin (Shi et al., 2017; Han et al., 2019). In contrast, miR-15b enhances cisplatin-resistant by targeting PEBP4- and RKIP-mediated EMT, as to miR-27a (Zhao et al., 2015; Yin et al., 2017). Interestingly, down-regulation of miR-199a-5p induces autophagy and re-sensitizes cells to multiple chemotherapeutic agents, nevertheless overexpression of miR-199a-5p inhibits autophagy and desensitizes cells to various chemotherapeutic agents (Zeng et al., 2021). In contrast, many miRNAs, such as miR-133a-3p, are regulates drug resistance in lung cancer patients by working with EGFR signaling networks (Li et al., 2021a). Upregulation of miR-762 induced by the IL-6 signaling pathway significantly increased cell survival and rendered NSCLC cells unresponsive to gefitinib-induced cell death (Ge et al., 2019). In addition, exosome miR-96 levels were not only significantly elevated in patients with radioresistant NSCLC, but were also significantly intimated with vascular invasion and poor overall survival (Zheng et al., 2021). In addition, microRNA-3127-5P can upregulate PD-L1, and the upregulation of PD-L1 induces immune escape and leads to chemotherapy resistance in lung cancer (Tang et al., 2018). These studies reflect the important role of miRNAs in drug resistance in NSCLC. A detailed study of the mechanism of miRNA drug resistance in NSCLC may provide new therapeutic targets. Next, we review the role of miRNA regulation in drug resistance in NSCLC (Figure 2).

## miRNA and drug resistance in NSCLC chemotherapy

Conventional chemotherapy remains the leading treatment for NSCLC patients, especially those with advanced stages. However, the rapidly development of resistance to chemotherapeutic drugs has greatly reduced the therapeutic efficacy of NSCLC patients. Numerous studies have identified several common signaling pathways, such as phosphoinositide 3-kinase (PI3K)/Akt/mTOR, MDM2/p53 and mitogen-activated protein kinase (MAPK)/Slug signaling, which are implicated in lung cancer chemoresistance (Xing et al., 2018; Li et al., 2021c). In addition to these pathways, some important mechanisms are also involved in chemotherapeutic resistance, such as 1) overexpression of efflux transporters (ATP binding box (ABC) transporter) (Choi and Yu, 2014), 2) defects in the apoptotic machinery (An et al., 2017), 3) activation of the EMT program (Erin et al., 2020), and 4) hypoxic environment and autophagy (Shahverdi et al., 2022). However, the mechanism of drug resistance is so intricacy that it has not been fully elucidated, and the interactions between these complicated mechanisms and their regulatory mechanisms remain largely unknown. To our interest, miRNAs play key roles in these biological processes. In terms of the role of miRNAs in chemotherapy resistance among these mechanisms, we describe the regulation of cisplatin, paclitaxel, and other chemotherapeutic agents (Table 1).

**TABLE 1:** Summary of miRNAs involved in drug resistance in chemotherapy of NSCLC.

MiRNAs	Drugs	Target	Mechanisms	References
miR-103A-3p	cisplatin	Bak1	inhibit apoptosis	Wang et al. (2021)
miR-10b	cisplatin	p53		Lin et al. (2021)
miR-197	cisplatin	Bcl-2, c-Myc, cyclin D1		Fujita et al. (2015)
miR-216b	cisplatin	Bcl-2		Vu et al. (2020)
miR-106a	cisplatin	ABCA1	increased efflux of drug and (or)	Ma et al. (2015)
miR-200b-3p	cisplatin	ABCA1	decreased drug intake	Liu et al. (2019)
miR-425-3p	cisplatin	AKT1	activate autophagy by negatively regulating the AKT/mTOR pathway	Ma et al. (2019)
miR-216b	cisplatin	PVT1	regulating apoptosis and autophagy through the Mir-216b/Beclin-1 pathway	Chen et al. (2019)
miR-19a	cisplatin	PTEN/Akt	inhibits CP and Akt by down-regulating PTEN	Xing et al. (2019)
miR-100	docetaxel	Plk1	influences cell proliferation, apoptosis, cell cycle distribution, and docetaxel sensitivity of LUAD cells	Feng et al. (2012)
miR-650	docetaxel	Bcl-2, Bax	Spc-a1 or H1299 cell colony formation was enhanced, which promoted cell growth	Huang et al. (2013)
miR-34c-5p	paclitaxel	Bcl-2	inhibit apoptosis	Catuogno et al. (2013)
miR-4262	paclitaxel	PTEN	through targeting PTEN and activating the PI3 K/Akt signalling pathway	Sun et al. (2019)
miR-421	paclitaxel	KEAP1 3′UTR	promoted the migration and invasion of lung cancer cells and inhibited apoptosis *in vivo* and *in vitro*	Duan et al. (2019)
miR-4443	epirubicin	INPP4A	regulating the activation of JAK2/STAT3 pathway	Zhang et al. (2018)
miR-199a-5p	doxorubicin	ABCC1, HIF-1α	increased efflux of drug and (or) decreased drug intake	Jin et al. (2020)

### Platinum resistance

Systemic therapy with cytotoxic drugs remains one of the primary treatment methods for NSCLC. Cisplatin is a commonly used chemotherapeutic drug and is an effective treatment for NSCLC when used in combination with other drugs (Teng et al., 2018). However, effective cancer treatment is hampered by patients’ resistance to cisplatin. Defects in the apoptotic machinery refer to cancer cells that can escape apoptosis and continue to survive either by overexpressing of anti-apoptotic proteins or underexpressing of pro-apoptotic proteins, and contribute to the development of drug resistance in NSCLC cells (An et al., 2017). However, miRNAs command the cell cycle by regulating proteins involved in DNA damage or inhibiting apoptosis-mediated cell death (Zang et al., 2017). Wang et al. found that cancer-associated fibroblast (CAFs)-derived exosomes miR-103A-3p promote cisplatin resistance by targeting Bak1 to inhibit apoptosis (Wang et al., 2021). One of the key regulators of apoptosis is the intrinsic mitochondrial pathway mediated by p53 (Hafner et al., 2019). As an upstream regulator of p53 signaling pathway, ectopic expression of miR-10b reduces the expression of p53 and its downstream effector factors and inhibits apoptosis, thus leading to cisplatin resistance (Lin et al., 2021). Bcl-2 family members, important anti-apoptotic proteins, are the most concerned miRNAs target molecules that promote cisplatin resistance in NSCLC through anti-apoptotic pathways (Su et al., 2014). Fujita et al. confirmed that an miR-197 was down-regulated in platinum-resistant NSCLC samples, leading to activation of various oncogenes (Bcl-2, C-myc and Cyclin D1), promoting chemotherapy resistance, tumorigenicity and lung metastasis (Fujita et al., 2015). Subsequently, it was reported that miR-216b increased the expression of Bcl-2 by reducing apoptosis and promoting chemotherapy resistance in NSCLC cells (Vu et al., 2020).

Epithelial-mesenchymal transformation (EMT) plays a momentous role in chemotherapy resistance in NSCLC (Adachi et al., 2020). Studies have shown that microspherule protein 1 (MCRS1) is negatively regulated by miR-129-1-3 p, and MCRS1 can induce EMT and cisplatin resistance by increasing the expression of miR-155 (Liu et al., 2014). In addition, downregulation of miR-181 and miR-1244 was observed in cisplatin-resistant NSCLC cells, on the one hand promoting the occurrence of EMT, and on the other hand promoting the growth, migration and metastasis of cancer cells (Li et al., 2015; Li et al., 2016).

The escape of tumor cell from chemotherapy can be achieved by regulating the expression of the number of transporter molecules for drug influx and efflux. Adenosine triphosphate (ATP)-binding cassette (ABC) multidrug transporters confer drug resistance (Balzer et al., 2022). Several studies have confirmed that miRNAs can take part in chemoresistance by upregulating the levels of ABC membrane transporters. Experiments have shown that in NSCLC cell lines, miR-106a targets the ABCA1 transporter, which induces cisplatin resistance (Ma et al., 2015). miR-200b-3p is another miRNA that targets the ABCA1 transporter, and its expression is upregulated in lung cancer tumor cells, thus driving cell drug resistance (Liu et al., 2019).

There are abundant studies have reflected the important roles of miRNAs and autophagy in the evolve and drug resistance of lung cancer (Jing et al., 2020). For example, AKT1 can be targeted by exosome Mir-425-3p, and Mir-425-3p activate autophagy by negatively regulating and controlling the AKT/mTOR pathway, ultimately give rise to resistance to cisplatin-induced apoptosis (Ma et al., 2019). PVT1 may act as a competitive endogenous RNA of miR-216b, regulating apoptosis and autophagy through the miR-216b/Beclin-1 pathway and inhibiting the sensitivity of cisplatin in NSCLC (Chen et al., 2019). In addition, inducing resistance to both Akt and CP *via* downregulation of PTEN is demonstrated by miR-19a (Xing et al., 2019). However, studies have found that intentional upregulation of miR-30a can improve the prognosis of NSCLC after neoadjuvant chemotherapy and inhibit drug-induced autophagy and drug resistance (Lin et al., 2021). So, miR-30a could be a target for effective chemotherapy and a monitoring marker. Direct targeting of miR-101-3p at ATG4D can upregulate the sensitivity of NSCLC cells to cisplatin, induce cell apoptosis, and inhibit autophagy (Cui et al., 2021). Therefore, miR-101-3p may also be a potential therapeutic target molecular for NSCLC treatment.

### Taxanes resistance

Taxanes, a first-line treatment for NSCLC, are commonly used in combination with other anticancer drugs for lung cancer (Long and Suresh, 2020). Resistance to paclitaxel is common in NSCLC patients. In Feng’s study, miR-100 downregulation was first reported in the docetaxel-resistant LUAD line SPC-A1/DTX compared with the sensitive parent cells SpC-A1 (Feng et al., 2012). It has also been proved that miR-100 directly targets Plk1 in SPC-A1/DTX cells, and low expression of Mir-100 can lead to overexpression of Plk1 and ultimately lead to chemotherapy resistance of docetaxel in human LUAD (Feng et al., 2012). Subsequently, Huang et al. (2013) believed that miR-650 caused docetaxel chemotherapy resistance in lung adenocarcinoma cells by regulating the expression of Bcl-2/Bax. In addition, miR-34c-5p confers paclitaxel resistance in NSCLC cells by working with the Bcl-2 modifying factor (Catuogno et al., 2013). miR-4262 enhances paclitaxel resistance in NSCLC cells by targeting PTEN and motivating the PI3 K/Akt signaling pathway (Sun et al., 2019). The high expression of miR-421 may at least partially explain paclitaxel resistance in lung cancer patients, and miR-421 induces paclitaxel resistance in the way of binding to KEAP1 3′UTR (Duan et al., 2019). Interestingly, the overexpression of miR-221-3p regulates the MDM2/p53 signaling pathway. Down-regulation of miR-221-3p decreased the sensitivity of A549 cells to paclitaxel, while up-regulation of miR-221-3p partially reversed the resistance of A549 cells to paclitaxel (Ni et al., 2021). Therefore, miR-221-3p may serve as an effective therapeutic target for paclitaxel treatment. Besides, miR-379-5p potentiated paclitaxel sensitivity through targeting TRIM65 in NSCLC (Guo et al., 2022). There is potential for miRNAs to be markers of taxanes resistance or response, and by using these markers, treatment decisions can be guided and the sensitivity of cells to taxanes can be restored.

### Resistance to other chemotherapy drugs

Other chemotherapeutic agents have also developed resistance to NSCLC treatment. For instance, miR-363-3p expression enhances gemcitabine resistance in NSCLC cells (Bian et al., 2021). Liang et al. (2019) believed that gemcitabine resistance in NSCLC is caused by the interaction of several miRNAs, such as LET-7D-5P, LET-7I-5p, miR-17-5p, and miR-23b-3p. Furthermore, overexpression of miR-4443 generated the resistance to epirubicin of NSCLC cells in the manner of targeting INPP4A and adjusting the activation of the JAK2/STAT3 pathway (Zhang W.et al., 2018). Among a variety of miR-199a-5p targets, chemotherapy resistance increases the expression of ABCC1 and HIF-1α. miR-199a-5p is participate in chemotherapy resistance of NSCLC *via* regulating the expression of ABCC1 and HIF-1α (Jin et al., 2020). However, miR-451A mitigated resistance of doxorubicin in lung cancer by targeting c-MYC and inhibiting EMT (Tao et al., 2020). These miRNAs mentioned above can be regarded as potential biomarkers for the predict of drug resistance in patients with NSCLC.

## miRNA and drug resistance in NSCLC target therapy

NSCLC patients with epidermal growth factor receptor (EGFR) -activated mutations are clinically common and often benefit from EGFR tyrosine kinase inhibitors (TKIs). Unfortunately, more than 50% of first—and second-generation TKIs clinical trial resistance cases are caused by secondary mutations (T790M) or tertiary mutation (C797S) (Sequist et al., 2011). Aside from the T790M mutation, a more common event is EGFR amplification, which occurs in10% of NSCLC patients who develop drugs resistance to these therapies (Sigismund et al., 2018). Similar to chemotherapy, both epithelial-mesenchymal transformation (EMT) regulates and interferes with apoptosis induced by EGFR-TKIs are also cause EGFR-TKIs resistance. In addition, activation of several signaling pathways, for instance PI3K/AKT/mTOR constitute key transduction cascades responsible for tumor cell survival, proliferation, invasion and metastasis. (Fumarola et al., 2014). Thus, in NSCLC with EGFR mutation, miRNAs mediate drug resistance of TKIs by activating PI3K/AKT/mTOR signaling pathway (Table 2).

**TABLE 2:** Summary of miRNAs involved in drug resistance in targeted therapy of NSCLC.

dslrMiRNAs	Drugs	Target	Mechanisms	References
miR-135a	gefitinib	PI3K/Akt	promoted cell growth and metastasis and activated the PI3K/AKT signaling pathway	Zhang and Wang. (2018)
miR-522-3p	gefitinib	PI3K/AKT	activating PI3K/AKT signaling pathway	Liu et al. (2020)
miR-200c-3p	gefitinib	c-Met	promotes EMT and inhibit apoptosis	Wang et al. (2020)
miR-103	gefitinib	Bcl-2	inhibit apoptosis and promote epithelial-mesenchymal transformation	Garofalo et al. (2011)
miR-203	gefitinib	Bcl-2		
miR-19a	gefitinib	c-Met	through targeting c-Met 3′UTR regulation and c-Met protein dependence	Cao et al. (2017)
miR-506-3p	gefitinib	EMT	inhibiting EMT	Haque et al. (2020)
miR-630	gefitinib	miR-630/YAP1/ERK feedback loop	persistent activation of ERK signaling *via* the miR-630/YAP1/ERK feedback loop	Wu et al. (2018)
miR-135	gefitinib	TRIM16	by targeting TRIM16 as a tumor promoter, it is involved in the inherent mitochondrial apoptosis, caspase and JAK/STAT pathways in NSCLC cells	Wang and Zhang. (2018)
miR-223	erlotinib	FBXW7	activation of Akt and Notch signaling pathways	Zhang et al. (2017)
miR-873	gefitinib	GLI1	induced cell proliferation	Jin et al. (2018)
mir-6253p	gefitinib	AXL	*via* activation of the TGF-β/Smad pathway and EMT in EGFR-mutant non-small cell lung cancer	Du et al. (2020)
miR-196a	gefitinib	GLTP	inducing cell proliferation and inhibiting cell apoptosis	Liu et al. (2022)
miR-326	gefitinib	IFNAR2	prostate cancer-associated transcription 6 (PCAT6) activates the miR-326/IFNAR2 axis	Zheng et al. (2022)
miR-323-3p	oximitinib	PI3K/Ak	PI3K-Akt signaling pathway	Janpipatkul et al. (2021)
miR-1468-3p				
miR-5189-5p				
miR-6513-5p				
let-7c	osimertinib	WNT1, TCF-4	reduced proliferation and invasion	Li et al. (2020)
miR-210	osimertinib	E-cadherin	promote vimentin expression	Hisakane et al. (2021)
miR-184/miR-3913-5p	osimertinib	RAS-MAPK/PI3K	ctivation or abnormal regulation by bypass pathways	Li et al. (2021b)

### Resistance of first-generation EGFR-TKIs

The first-generation EGFR-TKIs (include Gefitinib and erlotinib) are applied to the treatment of lung cancer. What cannot be ignored, however, is the blossom of resistance to these agent treatments, and several studies have shown that miRNAs are involved. For instance, miR-135a is associated with the lack of PI3K/Akt pathway activation and is involved in gefitinib resistance (Zhang and Wang, 2018). Liu et al. reveal that exosomes released from H1975 could induce resistance of gefitinib to PC9 *in vivo* and *in vitro* by activating the PI3K/AKT signaling pathway. However, up-regulation of miR-522-3p can enhance drug resistance in PC9 cells to gefitinib (Liu et al., 2020). Among patients treated with EGFR-TKI, those with high expression of miR-200c-3p had significantly longer progression-free survival (PFS) than those with miR-200C-3p in low expression. Silencing of miR-200C-3p in EGFR TKI highly sensitive cell lines increases drug gefitinib resistance (Wang et al., 2020). An evidence-based study reports that activation or overexpression of c-MET reduced the levels of miR-103 and miR-203 *in vitro*, which typically function as oncosuppressor miRNAs. Hence, these two miRNAs could cause acquired resistance to EGFR-TKIs in cases of c-MET gene amplification or overexpression (Garofalo et al., 2011). miR-19a downregulation promoted gefitinib resistance and EMT in gefitinib-sensitive NSCLC cells (Cao et al., 2017). Down-regulation of miR-506-3p promotes EGFR-TKI resistance by inhibiting EMT in NSCLC cell line (Haque et al., 2020). The miR-630/YAP1/ERK axis has also been reported to promotes TKI resistance in EGFR-mutated lung tumor cells. miR-630 downregulation promoted ERK activation through YAP1 upregulation, resulting in TKI resistance (Wu et al., 2018), and miR-135 down-regulates CDH1 and β-catenin and upregulates PD-L1 by targeting TRIM16, promoting drug resistance of NSCLC cells to gefitinib (Wang and Zhang, 2018). The AKT and Notch signaling pathways in erlotinib-resistant cells were stimulated and activated by up-regulated miR-223, and mir-223 was highly expressed in erlotinib-resistant HCC827 cells compared to that in parental cells (Zhang et al., 2017). It was found that inhibition of miRr-873 not only enhanced angiogenesis of NSCLC cells but also led to gefitinib resistance, because down-regulation of miR-873 dramatically trigger proliferation of gefitinib-treated PC9 cells, up-regulation of GLI1 subsequently (Jin et al., 2018). Recent data suggest that TGF-β1-induced EMT may be associated with gefitinib resistance mediated by the miR-6253p/AXL axis, which contributes to gefitinib-acquired resistance (Du et al., 2020). Recent studies have found that glycolipid transfer protein (GLTP) is a direct functional target of miR-196a, which can down-regulate GLTP and lead to gefitinib resistance. moreover, forced expression of miR-196a can also increases gefitinib resistance in NSCLC tumor cells by inducing cell proliferation and inhibiting cell apoptosis (Liu et al., 2022). In addition, IFN-α receptor 2 (IFNAR2) was identified as a downstream target of miR-326, reducing gefitinib resistance by inhibiting IFNAR2 expression. However, prostate cancer-associated transcription 6 (PCAT6) enhances gefitinib resistance in NSCLC *via* the miR-326/IFNAR2 axis (Zheng et al., 2022). Numerous studies have confirmed the important role of miRNAs in acquired gefitinib and erlotinib resistance and targeting these miRNAs may be an effective treatment option for lung cancer patients with first-generation EGFR-TKI-resistant.

### Resistance of second-generation EGFR-TKIs

Second-generation EGFR-TKIs is the preferred choice after the developed resistance to first-generation EGFR-TKIs in NSCLC. At present, afatinib is the most commonly used second-generation EGFR-tkis clinically, followed by nalatinib and dacomitinib (Tripathi et al., 2020). Afatinib is an efficient TKI that acts similarly to first-generation EGFR-Tkis, targeting EGFR mutations as well as secondary mutations, such as T790M (Tripathi et al., 2020). However, acquired afatinib resistance has also been observed in clinical use. Hashida et al. (2015) established an afatinib-resistant cell line, HCC827-ACR, from MET-amplified cell lines. Several kinds of afatinib-resistant cell lines like HCC827-ACR, show EMT characteristics, and epigenetic silencing of miR-200c and miR-200c promoter region methylation was observed in these cell lines (Hashida et al., 2015). Subsequent studies have shown that miR-34a mimics act synergistically with afatinib, rociletinib, or osimertinib in all EFGR mutant cells (Zhao et al., 2017). Optimal and consistently strong synergies were observed in a cellular model of acquired resistance (Zhao et al., 2017). Although miRNAs are rarely reported to be involved in drug resistance to second-generation EGFR-TKIs, some miRNA changes have been observed when afatinib is used to treat NSCLC cancer.

### Resistance of third-generation EGFR-TKIs

To date, third-generation EGFR-TKIs represented by osimertinib have been prescribed usually for T790M-positive patient (Cross et al., 2014). Unfortunately, patients typically develop resistance to osimertinib after 6–17 months treatment (Walter et al., 2013). Coincidentally, miR-147b was discovered to be the most upregulated miRNA in lung cancer cells resistant to osimertinib and EGFR mutations (Zhang et al., 2019). Janpipatkul et al. (2021) showed that four exosomal miRNAs (miR-323-3p, miR-1468-3p, miR-5189-5p, and miR-6513-5p) can not only be used as molecular biomarkers for the detection of oximitinib resistance in NSCLC patients, but also have the potential to distinguish oximitinib-resistant and oximitinib-sensitive NSCLC patients. In addition, in NSCLC cells with EGFR T790M mutation, leT-7C-mediated EMT leads to osimertinib resistance (Li et al., 2020). Exosome miR-210 may play a crucial part in the development of osimertinib resistance in the tumor microenvironment, and work as a therapeutic target to overcome this resistance in EGFR-mutant NSCLC. According to reports, exosomes from EGFR-mutated NSCLC cells induce EMT and drug resistance in osimertinib-sensitive cells *via* miR-210 delivery (Hisakane et al., 2021). In patients with EGFR mutations and T790m positivity, elevated expression levels of miR-184 and miR-3913-5p in serum exosomes are resistant to oximitinib by activating the RAS-MAPK/PI3K pathway (Li et al., 2021b). These studies also confirmed the important role of miRNAs in osimertinib resistance; therefore, targeting them may improve and restore the sensitivity of NSCLC patients with inherent and acquired resistance to third-generation EGFR-TKIs. The interactions between various miRNAs and complex signaling pathways during the process of third-generation EGFR-TKIs resistance in lung tumor cells are summarized above, paving the way for the introduction of miRNA-based biomarkers to detect the third-generation EGFR-TKIs response in lung cancer patients.

## miRNA and drug resistance in NSCLC immunotherapy

Immunotherapy (IO) has revolutionized the treatment landscape of NSCLC, particularly immune checkpoint inhibitors (ICI), including the T lymphocyte receptor CTLA-4 (cytotoxic T lymphocyte antigen 4), programmed death receptor 1 (PD-1), and PD-ligand 1 (PD-L1) inhibitors (Horvath et al., 2020). Despite striking clinical improvements, most patients end up responding poorly to ICI treatment because of the emergence of primary or secondary resistance. Currently, immunotherapy against the PD-1/PD-L1 axis is the first-line or subsequent treatment option for NSCLC patients and PD-1/PD-L1 antibodies, including pembrolizumab, nivolumab, atezolizumab, and durvalumab (Herbst et al., 2020). Similarly, the development of resistance to the PD-1/PD-L1 blockade is inevitable. The PD-1/PD-L1 blockade is intended to reinvigorate exhausted tumor-specific CD8 + T-cells, and the expression of CD8 + T cells is required for therapeutic response (Kurtulus et al., 2019). Some studies have indicated that Mir-934 can interact with circUSP7 in CD8+T cells, and the activity levels of Mir-934 will be inhibited by circUSP7, which finally caused the function of CD8+T cells be impaired so that immune escape in NSCLC (Chen et al., 2021). A key requirement for the PD-1/PD-L1 blocking response is that tumor-specific T cells in the patient are inhibited by PD-1, and PD-L1 expression in the tumor inhibits PD-1+ T cells at sites that require antitumor activity (Poggio et al., 2019). miR-3127-5p promotes the expression of PSTAT3 induced PD-L1, and immune escape induced by increased PD-L1 expression in lung cancer (Tang et al., 2018). A necessary condition for tumor cells to prevent immune escape is sensitivity to cytotoxic molecules produced by T cells. In a study by Zhu et al. (2020), the miR-505-3p axis can affect the primary functions of CD8+T cells, including cytokine secretion and cytotoxicity. The introduction of miR-505-3p may provide a new direction for immune resistance in NSCLC.

Nivolumab, however, which targets PD-1 for its function, is preferentially propitious to NSCLC patients with high tumor mutation burden (TMB). In short, upregulation of miR-320b and miR-375 was related to resistance in nivolumab treatment whereas the upregulation of some miRNAs predicted responsiveness to nivolumab treatment, such as miR-93, miR-138-5p, miR-200, miR-27a, miR-424, miR-34a, miR-28, miR-106b, miR-193a-3p, miR-181A and so on. (Costantini et al., 2018; Fan et al., 2020a). However, it has been found that anti-PD1 exposure of T cells promotes the aggregation of exosomal miRNA-4315. And miR-4315 can be used to determine the time cycle in which ABT263 therapy can effectively accelerate tumor cell death and bypass anti-PD1 resistance. Therefore, miRNA-4315 could be used as a biomarker for the development of anti-PD1 antibody treatment resistance (Guyon et al., 2020). Research on the resistance of miRNAs against PD-1/PD-L1 in lung cancer will contribute to the development of personalized combined immunotherapy.

## miRNA and drug resistance in NSCLC anti-angiogenesis therapy

Neovascularization is a prerequisite for tumor proliferation and metastasis; thus, anti-angiogenic agents are indispensable in treating NSCLC (Assoun et al., 2017). However, the emergence of impedance towards angiogenic blockers has been regarded as the main challenge in cancer treatment using the anti-angiogenesis strategy. VEGF is a major regulator of any kind of blood vessel growth in the human body and plays a role in angiogenesis (Altorki et al., 2019). Alterations in VEGF-dependent, non-VEGF pathways, and the interactions of stromal cell are the mechanisms of anti-angiogenesis therapy resistance (Haibe et al., 2020). However, the major angiogenic factors are regulated by miRNAs. Shi et al. (2018) suggested that up-regulation of miR-126 down-regulates VEGF-A and VEGFR2 through inactivation of VEGF-A/VEGFR2/ERK signaling pathway, demonstrating its significance in regulating angiogenesis in lung cancer. Exosome miR-3157-3p regulates the expression of VEGF/MMP2/MMP9 from endothelial cells *via* target TIMP/KLF2, therefore, promoting angiogenesis and increasing vascular permeability, leading to drug resistance (Ma et al., 2021). In A549 cells, exosome miR-497 can effectively inhibit the expression of tumor-related genes, such as VEGF-A, suggesting that exosome miR-497 may be involved in the regulation of anti-vascular resistance (Jeong et al., 2020). Currently, there are few reports on the anti-angiogenic treatment resistance of miRNAs in NSCLC. Due to the important role of miRNAs in angiogenesis, further research on the anti-angiogenic treatment resistance of miRNAs is needed to provide a new direction for the treatment of NSCLC.

## miRNA and radioresistance in NSCLC

Radiotherapy, a widely used and effective therapy in clinics, can help improve the 5-year survival rate of NSCLC patients. However, radiation therapy is not as effective as it could be due to the complicated genetic cellular responses to radiation. To date, there have been many reports on the relationship between miRNAs and radiotherapy resistance in NSCLC. Previously, upregulation of miR-95 was shown to promote radiation resistance in NSCLC cells by directly targeting Nexin1 (SNX1) (Chen X.et al., 2014). Yuan et al. (2020) showed that miR-410 was a vital regulator of EMT and radiation resistance in NSCLC, and induced EMT promoted the enhancement of radiation resistance by targeting the PTEN/PI3K/mTOR axis. MiR-22 is downregulated in NCI-466 SCLC cells and inhibits radiosensitivity *via* targeting Werner helicase-interacting protein-1 (WRNIP1) (Jiang W.et al., 2019). It has been reported that miR-183 expression could give rise to radiation-resistant in lung adenocarcinoma cell lines (H1299R cells), and miR-183 promotes EMT and radiation tolerance in H1299 cells (Huang et al., 2021). Fan et al. found that mTOR is a direct target gene of miR-1246 that mediates miR-1246-induced autophagy activation. MiR-1246 of intracellular and extracellular were found to be upregulated in a time-dependent manner after irradiation, giving rise to radioresistance in NSCLC cells (Fan et al., 2020b). Moreover, it was observed in Chen’s experiment, apoptosis induced by radiotherapy in the miR-181a mimic group was markedly inhibited in comparison with that of the miR-181a inhibitor group. also reported that miR-181A inhibits PTEN in non-small cell lung cancer and reduces radiosensitivity (Chen et al., 2020b). However, some miRNAs strengthen the sensitivity of NSCLC cells to radiation. For example, miR-9 can make cancer cells die by inhibiting the activity and migration of A549 cells and enhancing the radiosensitivity of A549 cells. This effect is highly regulated by the methylation state of its promoter, so the overexpression of miR-9 enhances the radiosensitivity of NSCLC (Wei et al., 2019b). In A549-R and H1299-R cells, miR-129-5p expression was markedly decreased, whereas SOX4 and RUNX1 expression was increased. MiR-129-5p was then transfected into NSCLC cell lines to caused cell apoptosis, DNA damage and cell cycle arrest, therewith restrain cell proliferation and colony formation by targeting RUNX1 and SOX, making A549-R and H1299-R cells sensitive to radiation (Xue et al., 2021). Potassium voltage-gated channel subfamily Q member 1 opposite strand 1 (KCNQ1OT1), is an imprinted antisense lncRNA located in the KCNQ1 locus on human chromosome 11p15.510, working in several human cancers (Sachani et al., 2018). In a study by HE (He et al., 2020), KCNQ1OT1 upregulation induced ATG5/ATG12-mediated autophagy through miR-372-3p, thereby promoting radiotherapy resistance in lung cancer. Therefore, miR-372-3p may also plays an important role in radiation resistance. Besides, using lung cancer cells, Sun et al. (2020) demonstrated that miR-125a-5p enhanced the radiosensitivity of these cells by upregulating SIRT7 and further increasing apoptosis. Taken together, using these findings, new directions can be taken to improve the radiosensitivity of malignant lung tumors.

## MicroRNAs delivery method in the treatment of NSCLC

MiRNAs have been the subject of several research studies since Lee et al. (1993) discovered them in 1993, and their function continues to be unraveled as they are discovered. It has become increasingly interesting to examine the way miRNAs are delivered in cancer therapy. The trial of Orellana et al. (2017) that the microRNAs are attached directly to folate (FolamiR), which allows them to be delivered directly into cells overexpressing the folate receptor, and using an autochthonous lung cancer model, they demonstrate that FolamiR-34a, a tumor-suppressor, is rapidly taken up by tumors and slows tumor growth. Afterwards, Perepelyuk et al., 2018 demonstrated that using MUC1-aptamer-functionalized hybrid nanoparticles, miRNA-29b could be efficiently delivered to NSCLC patients for downregulating target oncogenes. An early-stage lung cancer metastasis model showed significant tumor inhibition and survival enhancement with CL-PVAX-Mir-143 systemically delivered. It is important to note that the same results were obtained in advanced mouse models that had metastases. And there was no obvious acute toxicity associated with Cl-pvax-mir-143 treatment (Jiang Q.et al., 2019). Therefore, the delivery of Mir-143 is likely to be a breakthrough point in the treatment of NSCLC. According to two studies published recently, lung cancer development is suppressed by UTMD-mediated delivery of miR-216bM and miR-21-5p inhibitors (Wang et al., 2022; Zhou et al., 2022). Taking these examples into account, Deep dissection of miRNA delivery patterns will be a breakthrough in the journey of lung cancer treatment and a focus of scientists’ future research.

## Potential of miRNAs in the treatment of NSCLC

Among the benefits of miRNAs for clinical use include high stability in serum, fast noninvasive testing, and easy detection in tissues, blood, or other body fluids (Li and Sarkar, 2016). The similarities between miRNAs and their target genes, as well as the fact that a single miRNA can regulate multiple miRNAs, make miRNAs more likely to become biomarkers than mRNAs alone. Also, miRNAs regulate cancer cell proliferation, apoptosis, migration and tumor progression, making new lung cancer therapies possible. We also summarized the clinical trials for lung cancer treatment (Table 3).

**TABLE 3:** NSCLC clinical trials with miRNA-based therapy.

miRNA	drug	Population	Number	Toxicities	Safety (≥G3,%)	Efficacy	Status	References
miR-16	TargomiRs, MesomiR-1 trial (NCT02369198)	NSCLC,MPM	27	Infusion-related inflammatory symptoms, coronary ischemia, anaphylaxis, cardiomyopathy, non-cardiac pain	lymphopenia (42), increased AST or ALT (19), temporal hypophosphatemia (15), infusion-related inflammatory symptoms (8), cardiomyopathy (4)	ORR: 5%, SD: 68%, DOR: 32 weeks	Completed	Van Zandwijk et al. (2017)
miR-34	MRX34 (NCT01829971)	NSCLC,SCL,HCC,RCC,GIST, Melanoma	85	Hypoxia, neutropenia, thrombocytopenia	SAEs (35), lymphopenia (18), chills (14), deaths (9), fatigue (9), neutropenia (8), thrombocytopenia (6), back/neck pain (5), dyspnea (5)	ORR: 4%, SD for ≥4 cycles: 24%	Early closed	Hong et al. (2020)

HCC, hepatocellular carcinoma; RCC, renal cell carcinoma; GIST, gastrointestinal stromal tumor; SAEs, severe adverse events; ORR, objective response rate; SD, stable disease; MPM, malignant pleural mesothelioma; AST, aspartate aminotransferase; ALT, alanine aminotransferase; DOR, duration of the objective response.

Cancer cells use miRNAs for various functions, with some of them specifically targeting cancer-related pathways, posing a potential therapeutic opportunity. As part of the phase I MesomiR-1 study, TargomiRs, a miR-16-based microRNA mimic, was tested in those with malignant pleural mesothelioma or NSCLC at advanced stages in 2017. A newly developed anti-EGFR antibody, targeting EDVTM nanocells, was used in this trial to deliver MESOMir-1 to patients with EDVTM-targeted nanocells, and the first five patients were exposed to 1.5g of the phase 1 dose, which had an acceptable safety profile. In general, there has been a good tolerance and safety record for TargomiR treatment among patients (Van Zandwijk et al., 2017). Subsequently, in this phase 1 trial of microRNA-based cancer therapy, MRX34 (RP2D), a liposome analog (miR-34a), was identified and evaluated. Patients treated with MRX34 receiving premedication with dexamethasone showed manageable side effects and some clinical response. As a result of severe immune-mediated adverse events (AEs) that resulted in the deaths of four patients, the trial was closed early (Hong et al., 2020). Additionally, as the mechanisms by which they function are clarified, miR-200 and let-7 have been extensively used as therapeutic targets in animal studies, and they have been validated (Pecot et al., 2013; Stahlhut and Slack, 2015). It is important to note that miRNA-based therapies are still in the early stages of phase I clinical trials, but dose-dependent modulation of relevant target genes is proving that miRNA-based cancer treatments are feasible.

It might be possible to predict cancer response to chemotherapy or other therapeutic interventions using microRNAs. According to the study, 148 LUAD patients who had received pemetrexed maintenance therapy were negative for mutations in the EGFR or translocations of the ALK, compared with patients who were treated with pemetrexed, patients who expressed the levels differ of miR-25, miR-145, and miR-210 had significantly shorter progression-free survival times, this suggests that these three miRNAs can predict maintenance treatment efficacy (Mok et al., 2019). In addition, miR-125b-5p levels in partial response-post samples were significantly lower, monitoring miR-125b-5p levels in response to anti-PD-1/PD-L1 therapy might be useful (Liang et al., 2020).

There is growing evidence that miRNAs having a significant impact on predicting lung cancer outcomes. For example, identified miR-374a and miR-374b, two EMT-related miRNAs that may be associated with poor survival, after profiling miRNA expression levels in NSCLC patient samples (Kim et al., 2020). An analysis conducted recently found, the expression of miR-2355-3p in LUAD is upregulated, and this overexpression allows us to distinguish LUAD serum from normal serum. Additionally, the prognosis of patients with high miR-2355-3p levels is unfavorable. By targeting ZCCHC14, miR-2355-3p can be inhibited to suppress the progression of LUAD (Zhao et al., 2021). As such, miR-2355-3p may prove to be an effective noninvasive diagnostic and prognostic factor as well as a potential therapeutic target for lung cancer.

## Conclusion and prospects

In conclusion, the death rate of lung cancer is increasing year by year, and the existence of drug resistance is a major cause of treatment failure. A number of important roles that miRNAs play in cancer biology have been recognized in recent years, and they are one of the most exciting discoveries in the field of NSCLC. As oncogenes or tumor suppressor genes, miRNAs have been implicated in proliferation, apoptosis, angiogenesis, invasion, and migration of lung cancer cells. Drug resistance in lung cancer is also influenced by miRNAs in a variety of ways, but many miRNAs involved in drug resistance remain unknown. In addition, the analysis of miRNAs is potentially useful for lung cancer diagnosis, prognosis, and predictability, may provide a reliable, non-invasive biomarker for monitoring relapse and individual response to treatment. Therapeutic potential of miRNA mimics or miRNA inhibitors (such as anticancer drugs) could be achieved by mimicking tumor suppression of miRNAs. Despite this, certain challenges cannot be ignored. To start with, miRNAs are expressed heterogeneously, and their interactions with other miRNAs and effects on cell function are significant. It is essential to study the network of interactions between miRNAs and whether these molecules are complementary or antagonistic. A second point is that we still have very little knowledge about the mechanisms of miRNA secretion and uptake, and we need to further explore the specific molecules and mechanisms of novel miRNA delivery systems. In the third place, there is uncertainty about miRNA target selection and drug delivery accuracy. There is an urgent need for larger, more comprehensive studies to further optimize existing miRNA-targeted therapies and provide better therapeutic alternatives. Finally, there are few large-scale studies on the safety and efficacy of miRNA-based drugs, and reproducible results require studies involving stratified patients at large scale and ensure the clinical safety and efficacy of miRNAs as therapeutics. Overall further research on miRNA and its regulatory function will provide new ideas for preventing, monitoring, and treating lung cancer. To gain a deeper understanding of miRNA-based cancer treatments, future research is necessary.
